# Informing the Design of Direct-to-Consumer Interactive Personal Genomics Reports

**DOI:** 10.2196/jmir.4415

**Published:** 2015-06-12

**Authors:** Orit Shaer, Oded Nov, Johanna Okerlund, Martina Balestra, Elizabeth Stowell, Laura Ascher, Joanna Bi, Claire Schlenker, Madeleine Ball

**Affiliations:** ^1^ Human-Computer Interaction Lab Computer Science Department Wellesley College Wellesley, MA United States; ^2^ Department of Technology Management and Innovation School of Engineering New York University New York, NY United States; ^3^ PersonalGenomes.org Boston, MA United States

**Keywords:** genomics, genetic testing, interactive visualizations, personal electronic health records, direct-to-consumer genetic testing

## Abstract

**Background:**

In recent years, people who sought direct-to-consumer genetic testing services have been increasingly confronted with an unprecedented amount of personal genomic information, which influences their decisions, emotional state, and well-being. However, these users of direct-to-consumer genetic services, who vary in their education and interests, frequently have little relevant experience or tools for understanding, reasoning about, and interacting with their personal genomic data. Online interactive techniques can play a central role in making personal genomic data useful for these users.

**Objective:**

We sought to (1) identify the needs of diverse users as they make sense of their personal genomic data, (2) consequently develop effective interactive visualizations of genomic trait data to address these users’ needs, and (3) evaluate the effectiveness of the developed visualizations in facilitating comprehension.

**Methods:**

The first two user studies, conducted with 63 volunteers in the Personal Genome Project and with 36 personal genomic users who participated in a design workshop, respectively, employed surveys and interviews to identify the needs and expectations of diverse users. Building on the two initial studies, the third study was conducted with 730 Amazon Mechanical Turk users and employed a controlled experimental design to examine the effectiveness of different design interventions on user comprehension.

**Results:**

The first two studies identified searching, comparing, sharing, and organizing data as fundamental to users’ understanding of personal genomic data. The third study demonstrated that interactive and visual design interventions could improve the understandability of personal genomic reports for consumers. In particular, results showed that a new interactive bubble chart visualization designed for the study resulted in the highest comprehension scores, as well as the highest perceived comprehension scores. These scores were significantly higher than scores received using the industry standard tabular reports currently used for communicating personal genomic information.

**Conclusions:**

Drawing on multiple research methods and populations, the findings of the studies reported in this paper offer deep understanding of users’ needs and practices, and demonstrate that interactive online design interventions can improve the understandability of personal genomic reports for consumers. We discuss implications for designers and researchers.

## Introduction

### Overview

Recent years are seeing a dramatic growth in the availability of personal genomic data to end users. Consumers with varying levels of relevant education who seek genomic testing services are confronted with an unprecedented amount of sensitive information about themselves [1], often online and in interactive forms [2]. These consumers are not necessarily experts in genetics. They span the gamut from curious or concerned laypeople, to educated early adopters, to experts in genetics. Although genetic testing is available to these diverse populations, the resulting data reports can be difficult to understand without specialized training. Furthermore, the inherent complexity of genomic data is compounded by the frequency with which research in genetics is updated.

Consequently, questions about how consumers understand and engage with their personal genomic information are not only of paramount importance for society and policy makers, but are also a pressing issue for human-computer interaction (HCI) researchers. Specifically, the highly personal and dynamic nature of personal genomic information raises the following questions: What are the functional requirements for supporting meaningful engagement of consumers with varying levels of relevant knowledge with personal genomic information? How can we design effective interaction with personal genomic information? How can we evaluate the effectiveness of interactions with personal genomic information? Addressing these questions, this paper explores the roles HCI can play in helping consumers understand and engage with personal genomics.

We present findings from three complementing research activities:

1. Study 1: Understanding Users. Study 1 consists of a qualitative study with early adopters to understand users’ motivations, needs, and information practices when engaging with their personal genomic information.

2. Study 2: Informing Users. Study 2 involves a design workshop with early adopters in which the current state-of-the-art genomic reports are evaluated and various existing and possible features for interactive reports are explored.

3. Study 3: Probing Users. Study 3 designs and tests alternative interactive reports informed by the needs and practices identified in the prior qualitative studies. The designs, using different visualizations, were tested using online experiments with Amazon Mechanical Turk users to investigate how variations in interface design and data visualization affect users’ understanding of, as well as preference and attitude toward, online personal genomic reports.

Taken together, these studies contribute toward understanding and improving the ways people engage with and understand personal genomics information.

### Background

#### Personal Genomics

The Human Genome Project (HGP) published the full reference sequence of the human genome in April 2003. This international, collaborative research program, whose goal was the complete mapping and understanding of all the human genes, lasted 13 years and cost US $2.7 billion. The HGP DNA sequence is a composite derived from the DNA of several anonymous volunteers. The first individual's genome was sequenced in 2007. Since then, many more individuals have had their genome, or part of it, sequenced anonymously for research, but until June 2013, only about 500 individuals had ever had their full results returned to them [3].  The cost of sequencing a single human genome has dropped from US $2.7 billion in 2003 to about US $5000 in 2013, a cost drop far faster than the rate of Moore's law [3]. Decreasing sequencing costs and technological advances offer the promise of personalized medicine to the masses, with genomic information integrated into medical care to provide individualized risk assessment, tailored lifestyle change recommendations, and medications to reduce risk [4].

#### Online Interaction With Personal Genomics

The precipitous decline in the costs of DNA sequencing has led to widespread access of personal genomic data.  An increasing number of large-scale efforts, representing millions of people combined, are already underway. For example, the government of England recently announced their plan to sequence and return whole personal genomes to 100,000 British citizens by 2017.  In the United States, the Veterans Administration is pursuing an effort that aims to enroll 1 million veterans in a research study that incorporates genetic profiling.

At the other end of the spectrum from large centralized efforts, several companies currently offer services directly to consumers. For example, Illumina provides consumers (with prescription) genome sequencing services. Direct-to-consumer genetic testing (DTCGT) is a relatively new and developing online service, which enables individuals to acquire genetic information without the mandatory involvement of a health care provider by sending a saliva sample to a DTCGT company, at the cost of a few hundred dollars. To date, DTCGT does not typically offer whole genome or exome sequencing, rather, these tests use the single nucleotide polymorphism (SNP)-chip technique, which looks at thousands of very short sections of DNA known to vary across populations [1]. Results are delivered through online interactive reports. Several popular DTCGT services additionally offer interactive online reports of nonhealth-related information including traits and ancestry information (eg, AncestryDNA [5] and Family Tree DNA [6]). The service 23andMe [7] also provided risk assessment results for about 250 conditions, however, as of December 2013 the reporting of health-related information directly to consumers has been stopped while it is undergoing US Food and Drug Administration (FDA) review, which seeks to determine whether test results are accurate and are adequately communicated to, and understood by, consumers [8].

Traditionally, medical genetic testing targets individual loci and is performed for specific medical contexts (eg, when investigating a suspected genetic condition). Results are returned in a verbal process, mediated by a medical expert. The decreased cost of genome-scale tests combined with their application to an increasingly broad scope of individuals means the number of possible genetic test results has become several orders of magnitude larger than traditional context. While an expert advisor may remain as a part of the initial communication of data results, it becomes infeasible to present results in the same verbal manner. Thus, interactive computer-mediated presentation of this data to individuals has become a core aspect of giving individuals access to their genome-scale test results. For example, Illumina’s genome sequencing service provides initial genetic counseling upon the return of results, but data has also been returned to consumers digitally on an Apple iPad using the Illumina MyGenome app, which allows users to browse their genome, compare it to a reference genome, and review a health report that provides risk assessment for about 250 conditions.

Additionally, individuals will increasingly have ongoing access to extensive genetic test data. In the United States, patients now have a legal right to directly access clinical test data [9]. In addition, to date, all of the DTCGT services mentioned above also return raw genotyping data to users, who in turn can actively engage with their personal genomic data, for example, by learning about specific gene variants or conditions of interest. Indeed, consumers of genomic data have been observed transporting their data between services to capitalize on different features that allow them to engage more deeply with their data. For example, 23andMe users may export their data to AncestryDNA for genealogy, or to the Personal Genome Project (PGP) database—discussed in the proceeding section—to share with people of interest. Because this data is inherently digital, and because its interpretation gets updated frequently based on new research findings, we anticipate increased focus on the development of online interactive report methods that perform automatic reanalysis.

In summary, given recent advances in the field of personal genomics and rapidly declining sequencing costs, it seems inevitable that there will be vastly increased demand for individuals understanding their own genome-scale data and its health implications. The personal and complex nature of personal genomic information and users’ interaction with it raise important HCI questions.

#### Personal Genome Project

The Personal Genome Project [10] is a nonprofit organization that seeks to improve the scientific understanding of genetic and environmental contributions to human traits through the creation of a public genetic database of 100,000 volunteers [11-13]. Participants must be willing to share their genomic sequences, as well as health data, with the scientific community and the general public. The organization consists of sites spanning four countries. The longest running PGP site is based out of George Church’s Lab at Harvard Medical School. The Harvard PGP was established in 2005. It began with a pilot study of 10 fully identified individuals, known as the PGP-10, and slowly scaled up. Today, more than 4000 US citizens are enrolled in the project through a process of “open consent” [14] to publicly share their genomic information. We established a design partnership with the Personal Genome Project and are collaborating closely with its researchers.

#### User Perspectives on Personal Genomics

Little empirical data exists about the attitudes and motivations of people who have their genome sequenced and interact with their data [15]. Only a few studies have recruited DTCGT consumers who had actually received their own personal genomic information. In these studies, curiosity was mentioned as the participants’ primary motivation for undergoing genomic testing [15]. Most respondents wanted to learn more about themselves, were curious about their genetic makeup, or wanted to learn about individual genetic risk factors. Participants also stated that they would use information gained from the test to take personal responsibility for their future health [16]. Other themes included fascination with genealogy, contribution to research, and recreation [15]. Studies also identified several concerns among DTCGT users, including privacy, as well as the nature of the results and their future impact [17-20]. Only a small number of users around the world have had their entire genome sequenced and returned to them—500 as of June 2013 [3]—and to our knowledge, no studies have investigated the perspectives of such users. Further research is needed to understand personal genomic users’ motivations and concerns, information needs and practices, and the factors that impact willingness to share information.

#### Related Work: Human-Computer Interaction for Genomics

To date, little HCI research has focused on direct user engagement with personal genomic information. Lachance et al [3] examined the features of websites in which consumers can directly purchase and receive genetic testing without the mandatory involvement of a health care provider. Their findings indicate that most users would struggle to find and understand the important information on the majority of sites. Other efforts have considered user engagement with genomic and biological information more broadly, focusing mainly on novel interaction techniques for large biological datasets. For example, Shaer et al [21] have discussed opportunities and challenges for applying tangible and embodied interaction for discovery and learning of genomics. Kuznetsov et al [22] described a possible role for HCI in supporting the growing community of do-it-yourself biology (DIYbio) citizen scientists. Schkolne et al [23] developed an immersive tangible interface for supporting scientists in the design of new DNA molecules. Also, several tabletop systems have also been developed to explore interactive visualization of large biological datasets—DeepTree [24] and PhyloGenie [25] allow users to explore and learn phylogenetic trees. Most closely related to our work is G-nome Surfer [26], a tabletop user interface for collaborative exploration and learning of genomic information. This tool was not, however, designed to support consumers as they explore their own personal genomic data.

## Methods

### Study 1: Understanding Users

To gain insight into the information needs and practices of consumers interested in directly engaging with their genomic information, we conducted an exploratory qualitative study [27]. We recruited 63 study participants (29 women, 46%), aged between 21 and 71 with an average age of 47 (SD 14) from the Personal Genome Project volunteer community. This population of early adopters consists of users of various genetic testing services, who already spent time working with different tools available to explore their data, thus allowing us to understand existing information practices and needs of consumers, who use a range of genetic testing services. The interactions between these early adopters and their data provide a strong basis for exploring future data visualizations that appeal to a more diverse population.

Participants completed an online questionnaire consisting of 10 open-ended questions (see Table 1) about their engagement with personal genomics services and data. Response length averaged 252 words per user. We analyzed the data using content analysis methods. First-level codes were developed from preliminary review of the data by two independent coders and were then collapsed into advanced categories based on frequency. Categories were analyzed for the identification of themes. From this, we reported results regarding users’ information practices and needs.

**Table 1:** Open-ended questions from Study 1.

Question number	Questions from online questionnaire
1	What are the main reasons for your interest in exploring your personal genomic information?
2	What impact did your discoveries have on your life and attitude toward your health? Was there anything that you did, started doing, or stopped doing as a result of getting your personal genomic information?
3	What new or unexpected things did you learn as a result of genetic testing?
4	Did your discoveries lead you to social or formal interactions with other people and if so, who? For example, did you discuss your results with health professionals, family members, scientists, or support groups?
5	What websites and computational tools did you use for engaging with your personal (or your family’s) genomic information? How did you use these tools to learn from your data?
6	What features or applications could help you manage and learn even more from your (or your family’s) personal genomic data?
7	What are the main reasons for your decision to share your personal genomic information on PGP^a^?
8	What were valuable aspects of your experience exploring and sharing your personal genomic information?
9	What concerns do you have regarding exploring and sharing your personal genomic information?
10	Is there anything else you think we should ask you about your experience of engaging with your genomic data?

^a^Personal Genome Project (PGP).

### Study 2: Informing Users

In order to gain further insight into how users engage with, and learn from, their annotated personal genomic reports we conducted a qualitative study of personal genomics users. Participants were once again recruited from the PGP volunteer community. This population was chosen specifically because of their deep understanding of the data and tools available, and because they are likely to be first adopters of any new tool for personal genomics. This study was held as a workshop, which took place during the Genomes, Environments, and Traits (GET) conference, organized by the Personal Genome Project in Cambridge, Massachusetts, in April 2014.

The study focused on interactions around a specific genome reporting tool, GET-Evidence [12], which is an interactive personal genomic report provided to all PGP volunteers. We chose to study this particular tool since it is one of the most comprehensive gene variant reports available for consumers. Other direct-to-consumer genetic testing providers return information to users related to their traits and ancestry, but not a health-related report. The service 23andMe provided risk assessment results for about 250 conditions up until December 2013, when they suspended reporting of health-related interpretations while it is undergoing FDA review, which examines whether test results are accurate and are adequately communicated to, and understood by, consumers [8]. All of the direct-to-consumer genetic testing services also return raw genotyping data to users, which can be used to engage with the data beyond the commercial provider's reports, for example, by seeking information about specific gene variants or conditions of interest.

**Figure 1:**
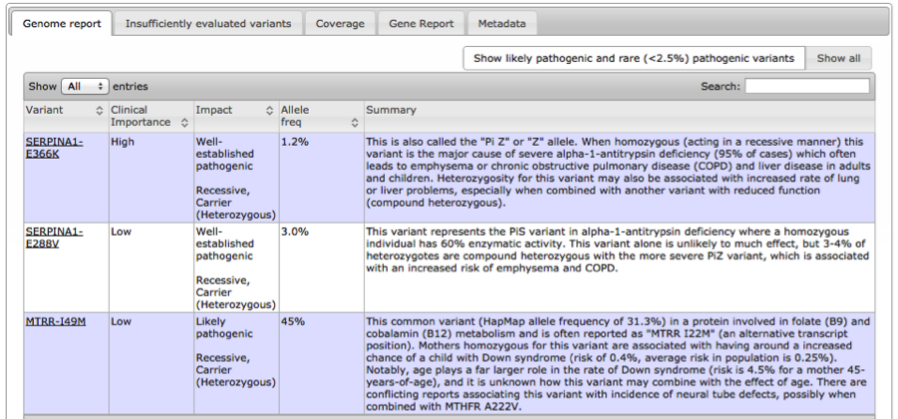
A screenshot of a GET-Evidence report, which utilizes tabular design.

The GET-Evidence report presents detailed information in a tabular design, including a list of gene variants reported to cause particular conditions or traits, the frequency of each variant in the population, the potential impact of each variant and the certainty of that impact (eg, well-established pathogenic, likely protective, uncertain benign), the clinical importance of each variant (ie, low, medium, or high), and a summary describing the current knowledge about a variant. Commentary and links to additional articles and external resources are also available. The table is sorted by clinical importance, but users can further sort their report based on the characteristics above (eg, by potential impact). Figure 1 shows a screenshot of a GET-Evidence report.

**Figure 2:**
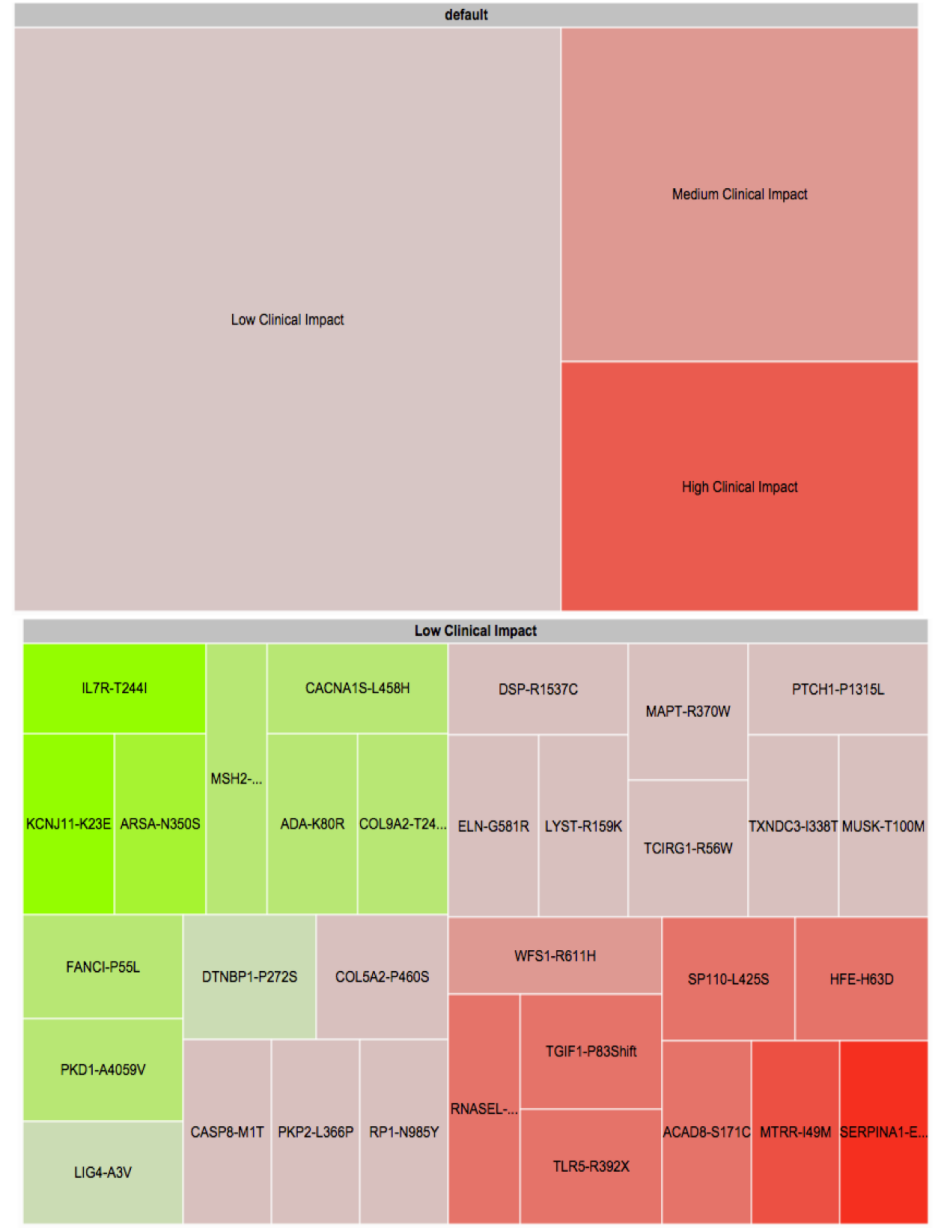
Two-level treemap prototype visualization of genetic variants. The top screen is the landing page for the visualization, whereas the bottom screen shows what happens when a higher-level rectangle is clicked on. Red represents pathogenic impact. Green represents protective impact.

Following a brief presentation that reviewed the goals of our research, 36 PGP volunteers—15 female (42%), aged 21 to 83 with an average age of 45 (SD 19)—were recruited to participate. We conducted in-depth, semistructured interviews with each participant. We asked users to explain their goals in engaging with personal genomic information, to share their information practices, and to show us how they use tools to learn from their data. We also asked participants to walk us through their workflow as they explore their personal GET-Evidence report (see Figure 1) [28]. Finally, to elicit ideas about new ways for visualizing and interacting with personal genomics, we presented users with a treemap visualization (see Figure 2) [29] of their own personal genomic data. Participants’ personal genomic data were retrieved from the PGP public database. We chose treemaps as a starting point for a discussion about new ways for presenting personal genomic data because they have been successfully applied to the visualization of gene ontologies [30]. Their application to personal genomics for use by consumers, however, is new. We asked users to compare the tabular report with the new visual report and to suggest further ideas that could improve their engagement with the data.

The prototype treemap visualization of the GET-Evidence report (see Figure 2) was created using Google Charts application programming interface (API). It presents the same information and interpretation as the original tabular GET-Evidence report. The treemap groups genetic variants by their clinical importance: low, medium, or high. Each variant is represented by a rectangle with a size proportional to its importance. The color represents the impact of a particular gene variant: pathogenic, benign, or protective. The saturation of the color represents the certainty of the scientific findings determining the impact of a gene variant where highly saturated colors represent high certainty. A red-green color scheme (red—pathogenic, green—protective) was used because it is well accepted in biology and is typically used for visualizing gene expression. Additional information about the gene variant, including a summary, is presented when hovering above a particular variant’s tile. Navigation between the two levels of the treemap is handled through selection.

Data were collected, included recordings of participant interviews, detailed notes, logs of user actions as they explored their data, and responses to an online questionnaire. Recordings were later transcribed and data were analyzed using content analysis methods by two independent coders.

### Study 3: Probing Users

#### Overview

Informed by the findings of Study 2, which will be discussed later and indicated that a visual summary of the report could potentially help nonexpert users in navigating and understanding personal genomic data, we conducted a between-subjects experimental study comparing the effects of different interactive visual genomic reports on nonexperts’ understanding of genomic data. These interactive reports were designed based on insights gained in Study 2.

**Figure 3:**
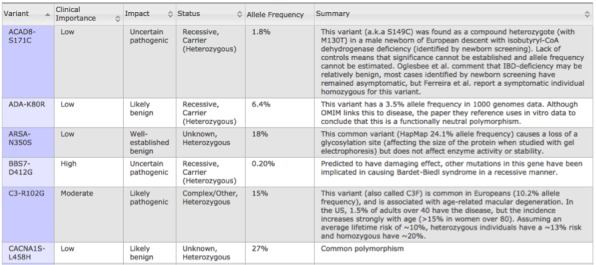
Table (control condition): gene variants are sorted by name. Variants can be further sorted by clicking on the arrows in each of the columns. The table is similar to the existing GET-Evidence report.

**Figure 4:**
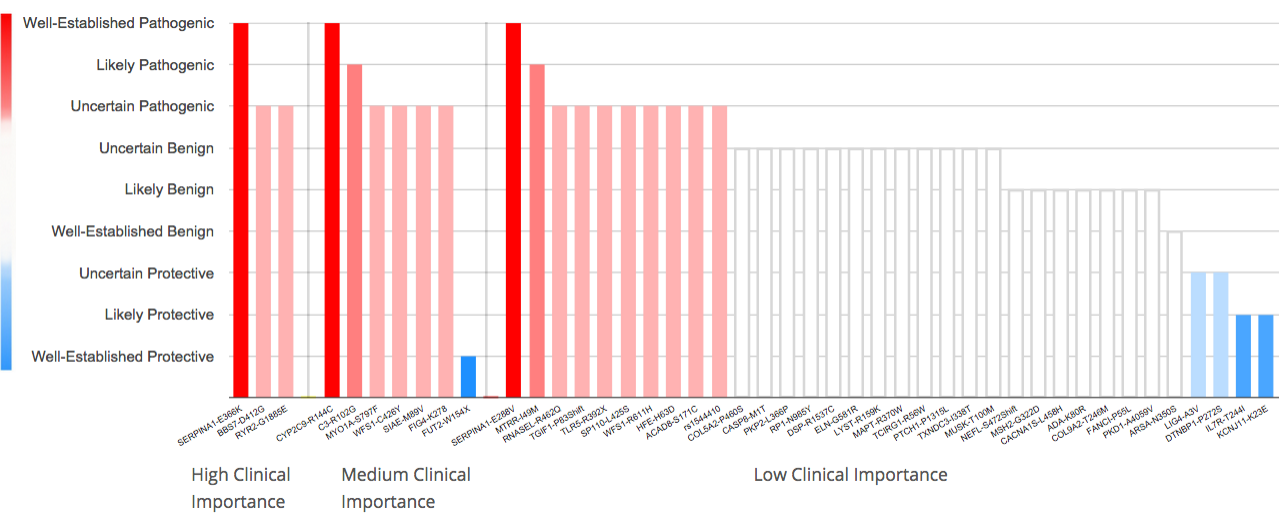
Bar chart: bars represent gene variants. A bar’s height and color represent the potential effect of the variant. Variants are separated by clinical importance. Information about a variant appears upon hovering over a bar.

**Figure 5:**
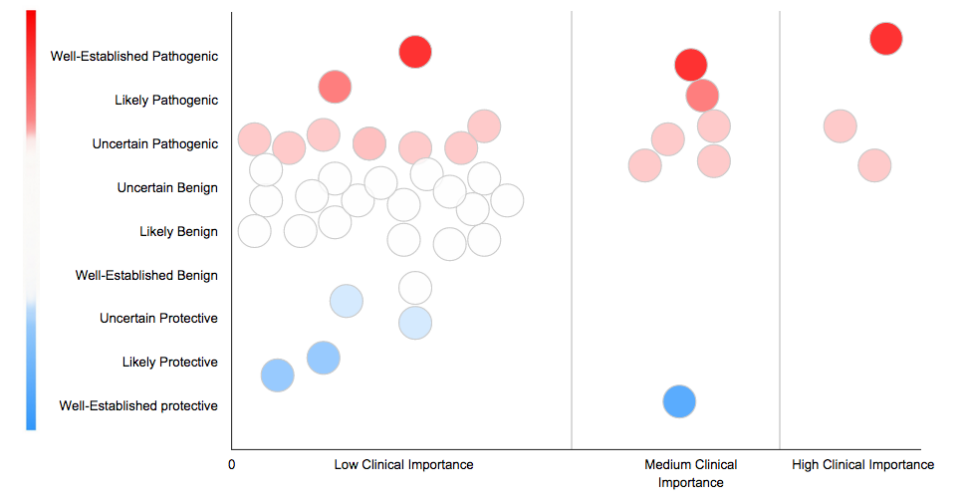
Bubble graph: each bubble represents a gene variant. A bubble's height and color represents the variant’s potential effect. Variants are separated by clinical importance. Detailed information about the variant appears upon hovering a bubble.

**Figure 6:**
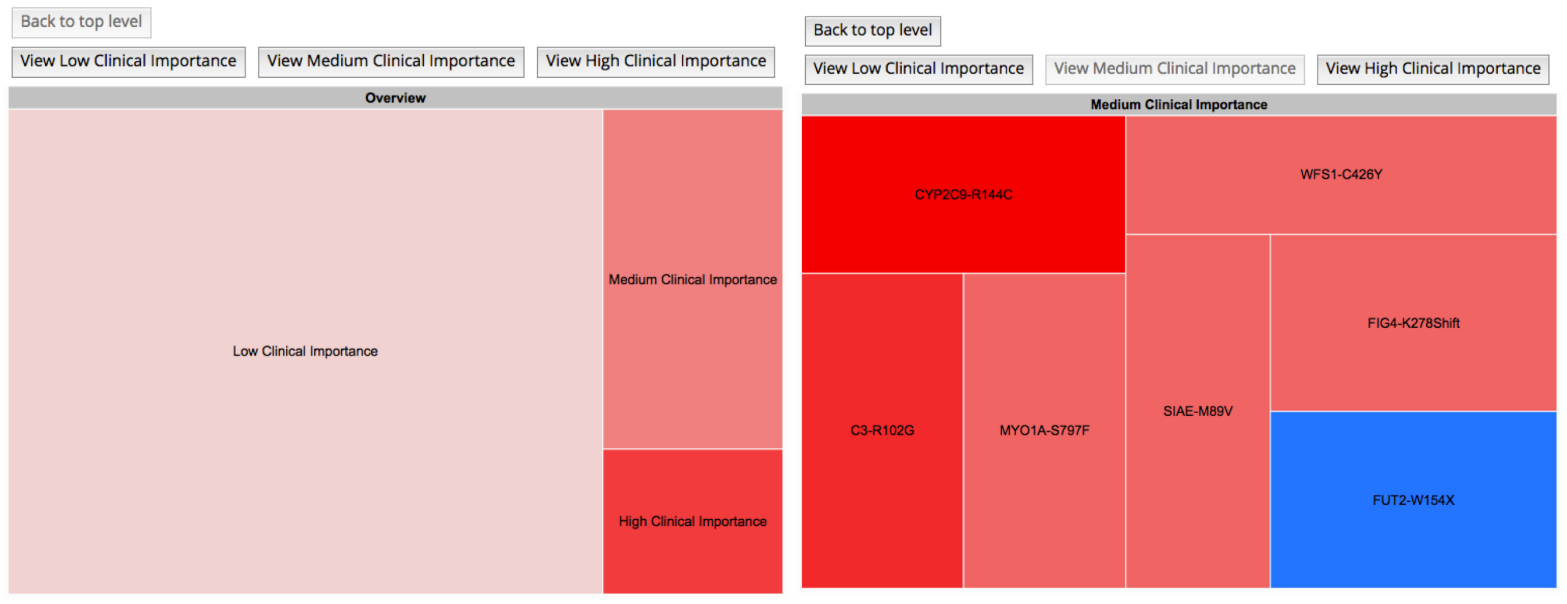
Treemap: gene variants are sorted by clinical importance. By clicking on the boxes or the buttons above the chart, variants within the clinical importance section selected appear and are color coded based on their potential effects. Detailed information about each variant appears upon hovering over it.

**Figure 7:**
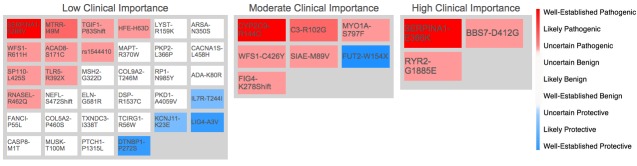
Heat map: each rectangle represents a gene variant. A rectangle’s color represents the variant’s potential effect. Variants are separated into the three gray boxes by clinical importance. Detailed information about the variant appears upon hovering over it.

**Figure 8:**
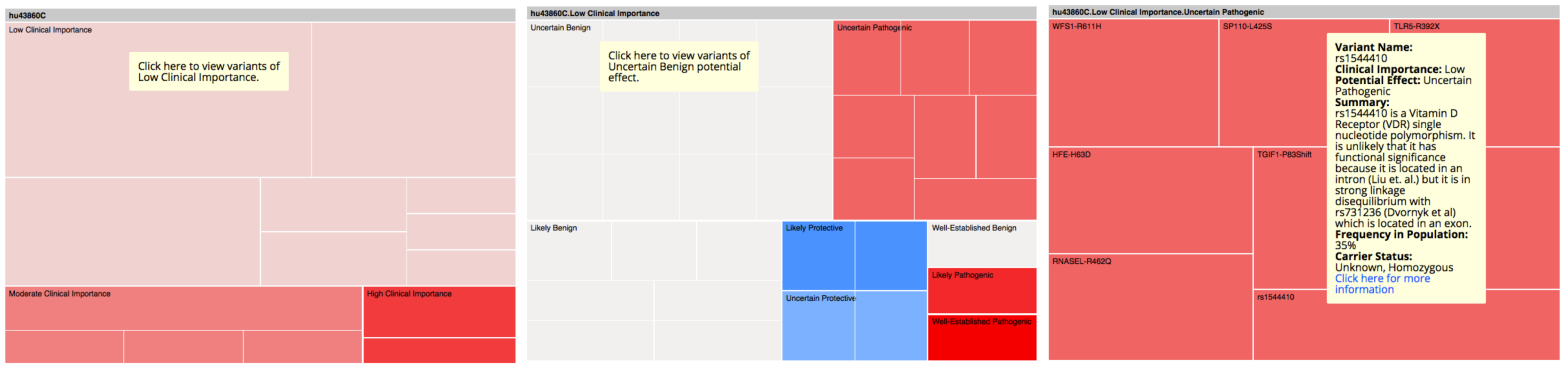
Zoomable treemap: the first level is sorted by clinical importance, the second level is sorted by potential effect, and the third level contains gene variants. The levels are navigable by clicking on the various rectangles. Detailed information about the variant appears upon hovering over it.

**Figure 9:**
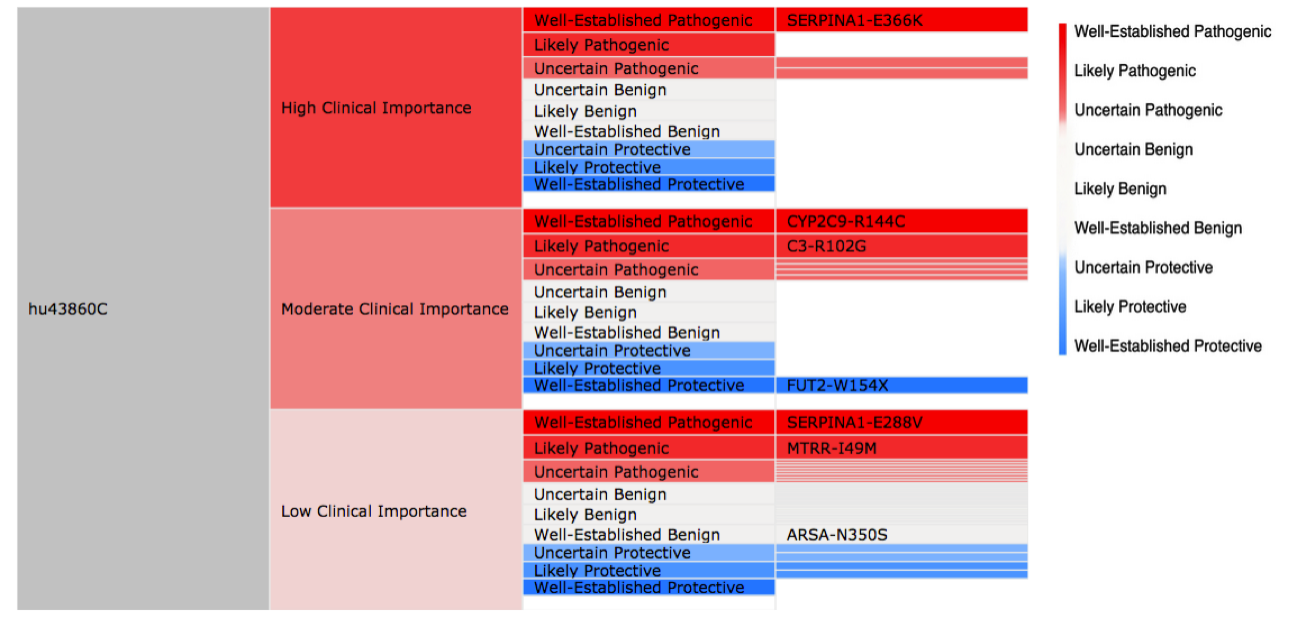
Zoomable partition: organized first by clinical importance, and then by potential effect. Zoom by clicking on the rectangles. Detailed information about the variant appears upon hovering over it.

An experimental website was developed specifically for this study, in which different versions of a personal genomics report using GET-Evidence interpretation (see Figures 3-9) were presented. The control condition for this study was a sortable table (see Figure 3), similar to the existing tabular GET-Evidence report. Genetic risk reports from other existing direct-to-consumer genetic testing services (eg, 23andMe) were not included in this evaluation, because they offer medical rather than genetic interpretation of the data.

We implemented the experimental Web platform using MySQL, PHP, JavaScript, Google Charts, and D3 libraries. Participants were recruited via Amazon Mechanical Turk and received US $1.00 for their time. Mechanical Turk is a crowdsourcing marketplace for online tasks that is widely used for HCI and medical informatics research [31-33]. We limited participation to US users with at least 100 prior Human Intelligence Tasks (HITs) at 99% or higher approval rate.

Participants first received a tutorial on the human genome and personal genomics using materials developed by the Personal Genetics Education Project [34]. Their understanding of the material was assessed through a short six-question quiz. If the participant was unable to answer at least three out of six questions correctly, their data were not used in the analysis. They were then presented with one of seven versions of the GET-Evidence report developed for this study.

#### Experimental Conditions

We developed six alternative designs of interactive visual personal genomics reports based on the user requirements and feedback solicited in the previous two studies. In particular, we focused on three of the functional requirements identified in Study 1 (as discussed in the Results section): reviewing an annotated report (R1), integrating data resources (in particular, summary of, and links to, scientific literature) (R2), and making content accessible to nonexperts (R6).

The interventions (ie, interactive reports) developed varied in the visualization technique used for a visual summary, and in the interaction techniques provided for exploring the data. Figures 3-9 show the seven experimental conditions: a tabular control condition (Figure 3) and six visual interactive reports.

Based on our findings from Study 2 that are discussed in more detail later on, we used a new red-white-blue color-coding scheme across all visual conditions to represent the impact and certainty of a particular gene variant. Color represents the impact—pathogenic (red), benign (white), or protective (blue). Saturation represents the certainty of the scientific findings determining the impact of a gene variant, where highly saturated colors map to high certainty. We also added a clickable glossary to all of the reports.

Participants were randomly assigned to one of the following seven conditions: a tabular report modeled after the GET-Evidence report (the control condition) (Figure 3, n=105), a bar chart (Figure 4, n=103), a bubble graph (Figure 5, n=115), a treemap (Figure 6, n=102), a heat map (Figure 7, n=104), a zoomable treemap (Figure 8, n=96), or a zoomable partition (Figure 9, n=105).

We used the same personal genomics data across the different versions, allowing for direct comparison of the reports. This approach of using a fictional dataset to assess user comprehension is a common practice in studies of personal genomics, for example, as in Haga at al [35] and Kaufman et al [36]. We chose a fictional dataset in which sex and ethnicity do not have a specific effect. Actual personal genomic reports include information regarding sex and ethnicity as it may have an effect on particular variations. Once participants had viewed the mock genome reports, they were asked to answer two types of questions: (1) comprehension questions which measure the effectiveness of the interactive visualizations in conveying genomic information, and (2) subjective questions on the extent to which users perceived the report to be understandable. Participants also responded to open-ended questions soliciting their perspectives on useful features and areas for improvement.

#### Visualization Questions

Participants were asked to answer a number of questions testing their understanding of the genomic information based on the visual report they were assigned (see Table 2, Q1 to Q9). The comprehension questions were written in collaboration with the Harvard Personal Genome Project's director of research. Participants also answered questions about their perception of the information (see Table 2, Q10 to Q18) and reported basic demographic information. Participants’ responses were recorded in a database and their performance and opinions were compared across the different experimental conditions.

**Table 2:** Understanding and opinion questions from Study 3.

Question number	Question	Question type
Q1	The number of variants with high clinical importance: _______	Fill in the blank
Q2	The number of variants that are well-established pathogenic: _______	Fill in the blank
Q3	Based on the information above, the number of variants in Jamie's report with low clinical importance is ________ the number of variants with high clinical importance.	Multiple choice: Greater than, Equal to, Less than, I don’t know
Q4	Based on the information above, the number of uncertain pathogenic variants in Jamie's report is ________ the number of well-established pathogenic variants.	Multiple choice: Greater than, Equal to, Less than, I don’t know
Q5	Based on the information above, the number of potentially pathogenic variants in Jamie's report is ________ the number of potentially benign or protective variants.	Multiple choice: Greater than, Equal to, Less than, I don’t know
Q6	Which variants would Jamie be most likely to discuss with a health care provider?	Open response
Q7	Based on the information above, Jamie's risk of developing stomach flu is ________ the average person.	Multiple choice: Greater than, Equal to, Less than, I don’t know
Q8	Based on the information above, Jamie's risk of developing age-related macular degeneration is ________ the average person.	Multiple choice: Greater than, Equal to, Less than, I don’t know
Q9	If you were Jamie, knowing this information, which of the following conditions would you be interested in learning more about? Select all that apply.	Checkbox: Alzheimer's, Parkinson's, Liver Disease, Colon Cancer, Diabetes, Emphysema, Tuberculosis, Eye Disease
Q10	The information in the report was presented in an accessible manner.	Likert scale
Q11	The report is easy to understand.	Likert scale
Q12	Jamie's genes determine everything about them and their future.	Likert scale
Q13	If I were Jamie, I would need the help of a health care professional to understand the results in the report.	Likert scale
Q14	The scientific knowledge used to generate this report is well established.	Likert scale
Q15	If I were Jamie, I would show the results in the report to my doctor.	Likert scale
Q16	The report gives me a firm grasp of Jamie's health and biology.	Likert scale
Q17	Please use the space below to tell us which features were most helpful for understanding the report.	Open response
Q18	Please use the space below to tell us how we can improve the report to make it easier to understand.	Open response

## Results

### Study 1

#### Demographics

A total of 83% (52/63) of the participants held academic degrees, 32% (20/63) held doctoral degrees, and 30% (19/63) worked in life sciences-related fields. This demographic is consistent with the description of early adopters provided by Rogers’ theory of the diffusion of innovations [37]. Early adopters tend to have advanced education, expert knowledge—though not necessarily in the subject matter at hand—and a willingness to engage in trials of new technologies.

All participants had prior access to their personal genomic data. Some users received genomic data from more than one service. Table 3 describes the genetic testing services used by study participants.

**Table 3:** Personal genomic data sources (n=63).

Personal genomic data sources	Users, n (%)
23andMe	38 (60)
Complete Genomics	11 (17)
Family Tree DNA	11 (17)
AncestryDNA	5 (8)
NG Genographic	2 (3)
Microbiome	2 (3)
Sorenson	1 (2)
Medical tests	1 (2)
Exome	1 (2)

#### Existing Tools

Participants were asked about the websites and computational tools they use to engage with their personal genomic information, and how they use these tools to learn from their data. We found that about 11% (7/63) of participants have used tools beyond those offered by their genetic testing service. An additional 10% (6/63) of participants had tried to explore their genomes using the tools provided by their service provider but found the tools to be too complicated, confusing, or “not user friendly.” Table 4 lists the tools and websites used by our study participants and highlights the main features of each tool.

**Table 4:** Interactive tools for exploring personal genomics (n=63).

Tool	Users, n (%)	Main features
23andMe	22 (35)	Testing service and interpretative tool Health report on over 240 conditions with external links (until December 2013) Visualized ancestry information Download of raw data file
Promethease	8 (13)	Interpretative tool Annotated report with latest information from SNPedia Comparison of two genomes Family report
SNPedia	5 (8)	Database Medical, genealogical, and phenotypic variant associations SNP^a^ articles with links to publications and summary Facility for sharing data Access to shared data
Personal Genome Project	7 (11)	Testing service and database Annotated report of variants sorted by clinical importance and impact Article for each variant includes summary and links to relevant publications Facility for sharing data Access to shared data
Gedmatch	7 (11)	Database and interpretative tool Comparison of user’s data with public results Visualized information about selected matches Genetic distance calculator Relationship calculator
AncestryDNA	7 (11)	Testing service and interpretive tool Information on ancestry Updates of new matches Access to family tree Download of raw data file
PubMed	6 (10)	Database Access to e-books and journals about all aspects of medicine and life sciences

^a^Single nucleotide polymorphism (SNP).

#### Functional Requirements

While participants reported being motivated by a diverse set of questions ranging from learning about their traits, to identifying health risks, to learning about their ancestry, they used existing tools to perform six common information tasks: reviewing an annotated report, integrating resources, curating information, comparing genomes, sharing information, and making content accessible. Table 5 depicts these information tasks, and provides an example quote motivating each task.

These tasks constitute the functional requirements for new interactive systems designed for direct consumer engagement with personal genomic information:

1. Task R1: reviewing an annotated report. Participants described the difficulty of interpreting existing tabular and dense textual reports. They expressed a desire for visualizations that make the information easier to explore and understand.

2. Task R2: integrating resources. Participants expressed a need for integrating various data resources, including annotated genomes, scientific publications, various public databases, and health-related data, into a single tool.

3. Task R3: curating information. Participants articulated a need for collecting, relating, organizing, and storing diverse information artifacts (eg, scientific papers, popular articles, notes for doctor appointments, gene variants, and videos) found throughout their independent research of their genome.

4. Task R4: comparing genomes. Participants asked for the ability to triangulate data from several individuals in order to understand connections within families.

5. Task R5: facilitating sharing information. Participants highlighted a need for tools that facilitate information sharing with family, friends, and genetic researchers.

6. Task R6: making content accessible. Participants indicated a need for adapting the content and language of personal genomic reports toward nonexperts. Users also asked to integrate educational materials within the reports and to point to actionable information.

**Table 5:** Information tasks and relevant quotes from users.

Task number	Task	Quote
R1	Reviewing an annotated report	“I'd be interested in seeing a graphic illustration of my chromosome sets.” “It would be great to show the SNPs^a^ by chromosomal location and in relation to other genes.”
R2	Integrating resources	“Integrated databases of published research that allow the end user, through a seamless interface, to connect personal data with any possibly relevant literature and public data.”
R3	Curating information	“Features that show more clearly what reasonable actionable options there might be for dealing with or preventing various illnesses.”
R4	Comparing genomes	“...easy to use, at home programs, will be needed to compare one's data with those of friends.”
R5	Facilitating information sharing	“The thing that would help the most would be for people to be willing to share more information.”
R6	Making content accessible	“Every time I try to understand something, I have to educate myself via Google, instead of the interface that gives me my genetic data educating me. The research it takes holds me back from using my info more.”

^a^Single nucleotide polymorphisms (SNPs).

### Study 2

#### Demographics

**Figure 10:**
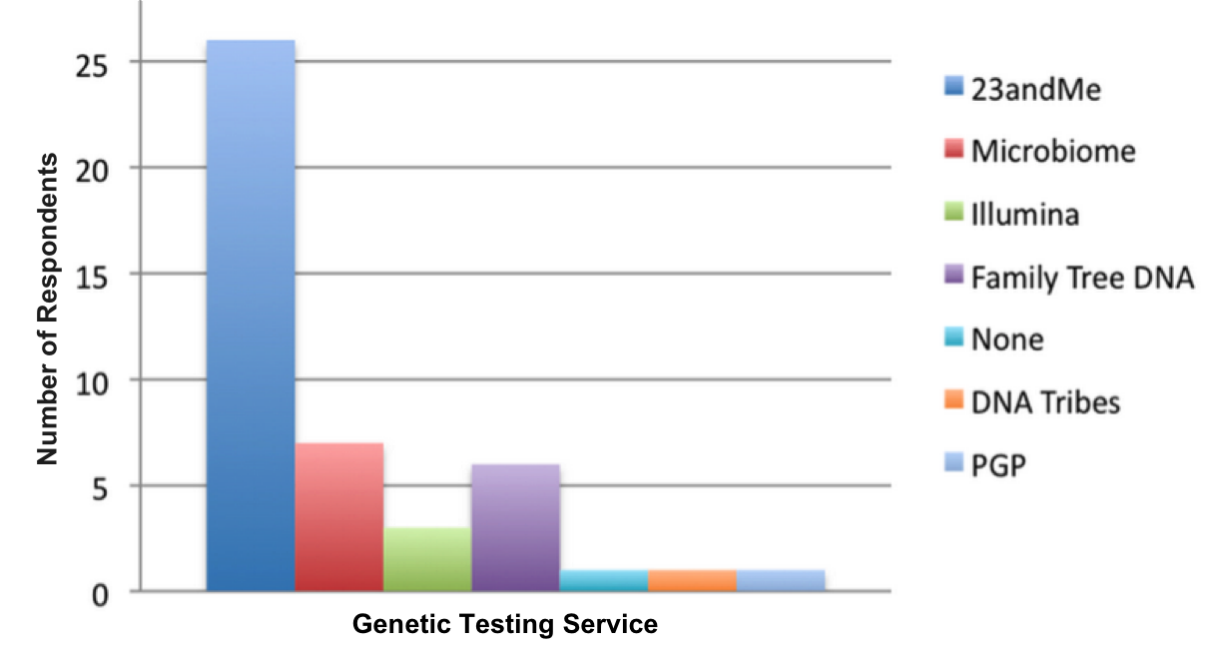
Genetic testing services used by study participants.

A total of 88% (32/36) of participants held academic degrees, 31% (11/36) held doctoral degrees, and 47% (17/36) worked in life sciences-related fields. All users had previous access to their testing service’s personal genomic report (eg, 23andMe report). Figure 10 demonstrates the distribution of personal genetic testing services. A total of 11% (4/36) of users first viewed their results within the 2 months prior to the study, 6% (2/36) within 3 to 6 months, and 83% (30/36) received the results more than 6 months prior to the study. Approximately one-third of participants had previous access to the GET-Evidence report generated by the PGP. Two-thirds of the users reviewed their GET-Evidence report for the first time in the workshop.

#### Motivation and Impact

**Figure 11:**
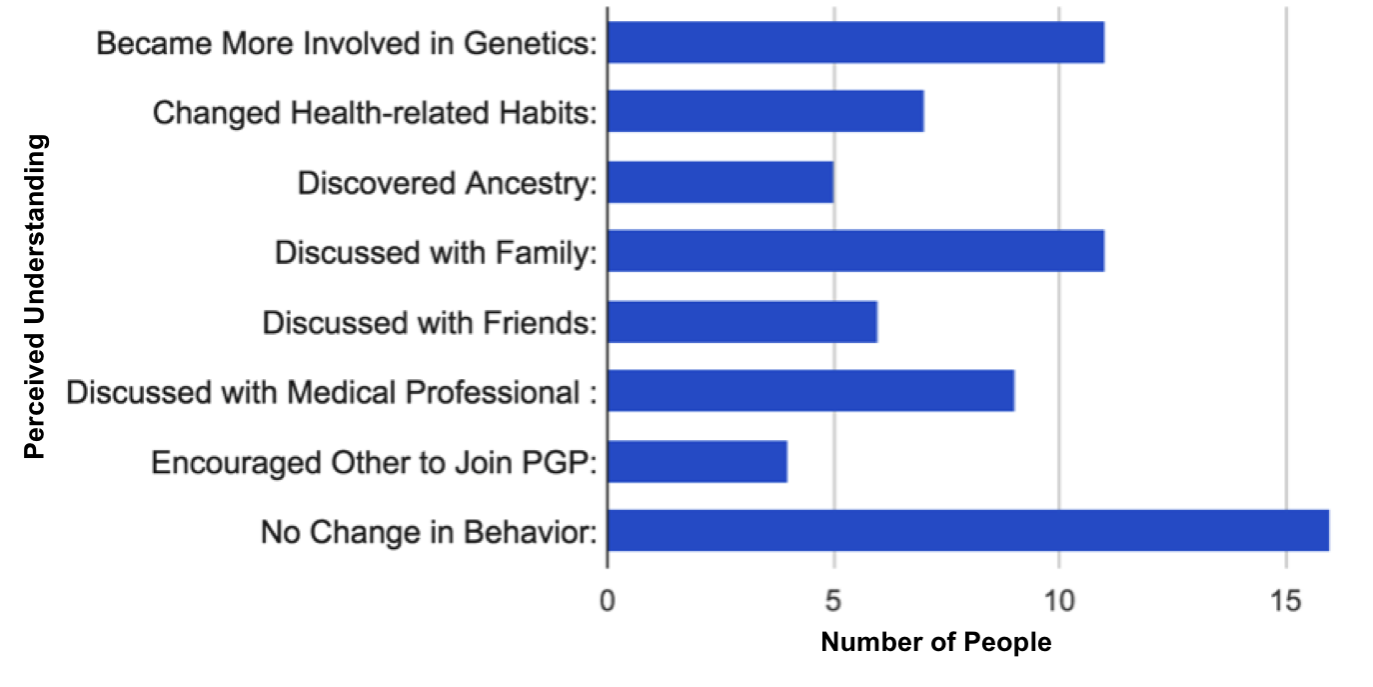
Reported impact of users' personal genomic information on their lives. Multiple users listed more than one influence.

Most participants provided more than one reason for exploring their genetic data, including understanding family and individual health risks, gaining insight into ancestry, satisfying curiosity, advancing science, and promoting open-source science. Specifically, 36% (13/36) listed understanding health risks as the primary reason for exploring personal genomics information, while over 66% (24/36) listed advancing genetic, scientific, and health research as their main reason. A total of 14% (5/36) noted that promoting open-source data was a motivation, and 19% (7/36) of users mentioned curiosity. Participants were also asked to describe how knowledge of their genetic traits and health risks impacted their lives. Figure 11 shows their responses. Multiple users listed more than one aspect of influence.

#### Tabular Report

Users spent about 10 minutes exploring their tabular GET-Evidence report. Most users began by reviewing the table and attempting to sort it according to the impact of the various variants. Users prioritized variants that have well-established pathogenic impact with high clinical importance. One explained, “Most interesting for me is what is high clinical important—I zoom in on things that say high, pathogenic, well-established.”

Many users commented on the amount and nature of information presented: “To me, it seems clunky and more like raw data, and could use some more sorting,” and “Show me the most important vectors vs the entire shebang.” Another user commented, “This is overwhelming, I prefer it filtered by high importance.” Users requested more advanced sorting and filtering mechanisms: “Being able to sort the list, so that you can see all pathogenic mutations together, or the mutations ranked by how well-studied they are.” A total of 4 users did not realize that the table could be sorted at all and scanned the table entries individually in search for important variants: “It was difficult scanning rows.” Some users requested direct search functionality that includes the detailed summary entries.

#### Visual Report

In order to initiate a conversation about alternative ways for presenting personal genetic information, we asked users to explore their own data using a treemap report (see Figure 2).

Users spent about 10 minutes exploring their data using the treemap report followed by a semistructured interview about the strengths and weaknesses of interactive visual representations of genetic data.

Approximately one-third of the participants expressed a strong preference for the treemap visualization. In the words of one participant, “I like this better in every way. It provides quick visual summary and weights the low and moderate by size so I can quickly determine what to be concerned about if anything.” Many of these users found the color coding to be particularly helpful: “The color shading made it easy to tell which alleles were protective or pathogenic.” Others suggested the inclusion of a color key, the use of a color scheme accessible for users with red-green color deficiency, and better color distinction between benign and pathogenic variants.

On the other hand, many users commented on the treemap navigation either finding it confusing or preferring to see all the information at once: “I dislike navigating because [I] need to do additional actions to access desired browsing criteria.” While we attributed some confusion to the navigation mechanism implemented by the Google Visualization API, we also identified the importance of balancing the requirement to highlight important information with the need to present to users an overview of the entire dataset.

#### Accessibility for Nonexperts

Users requested “nonscientist-friendly” reports that provide access to glossaries and use nontechnical language. About half of the users commented on the technical jargon used in the reports, finding it difficult to understand: “As a person that doesn’t understand science, it’s overwhelming.” In particular, several users asked about the following terms: allele frequency, homozygous, pathogenic, and benign. Some users noted that variant names, which are used as labels, are too long and intimidating for nonscientists, and suggested adding information beyond scientific variant names.

#### Complexity and Uncertainty

We learned that one of the most challenging aspects of presenting personal genomic information to consumers is the complexity and uncertainty of the interpretation. Associations between gene variants and medical conditions or traits are established through scientific studies that vary in the strength of the evidence they present. The GET-Evidence report classifies evidence into three main categories: established, likely, and uncertain. Multiple users thus commented on the challenge of understanding the potential impact and clinical importance of some variants. For example, one user asked, “Are variants with low clinical impact just variants that have not been well characterized yet?” After using the treemap report another user noted, “So much inadequate evidence out there—successful that it communicates that.”

In addition, many complex conditions such as diabetes or various cancers are associated with multiple genes rather than by a single gene variant. One participant requested, “A better understanding of how the factors combine to affect me.” Another user suggested, “It would be helpful to see similar diseases grouped together. For instance I had one protective and one negative SNP for macular degeneration, and it would be hard to connect that only looking at this report.” Furthermore, an individual may be a carrier for a certain trait, meaning that she or he is not impacted by particular gene variants but their children might be. As one user explains, “I am also interested in alleles that may not have an impact for me (as a carrier) but that could affect my future children if they end up getting two copies, so it would be nice to have a separate report that shows those.”

#### Providing Evidence

Several users requested that the reports provide direct links to sources of scientific data while grading the rigor of the studies: “Include links to relevant studies—back it up.”

#### Relating Genetic Variants to Medical Conditions

Finally, 6 participants out of 36 (17%) suggested focusing the results report on medical conditions rather than gene variants: “It would be helpful to have the health condition rather than the variant/trait displayed. [I] would rather see [medical] condition not variant at the tile label.” Participants also requested information on how to mitigate the risk for particular conditions to which they are genetically predisposed. However, in December 2013, the US Food and Drug Administration ordered companies that offer personal genome testing, such as 23andMe, to cease providing such health reports to customers [8]. The FDA was concerned with the use of direct-to-consumer genetic tests for medical purposes because of the uncertainty and inaccuracy of these tests in predicting disease risk. As a result, new medical interpretation of an individual’s genomic data, as requested by several participants, is beyond the scope of our investigation.

### Study 3

#### Overview

A total of 745 participants were recruited for this study. Participants whose post-tutorial test included three or more incorrect responses (out of six) were excluded from the analysis. The sample used in the analyses thus came to 731 individuals whose average age was 36.6 years (SD 11.9). A total of 40.6 % (297/731) of the participants were women, 56.8% (415/731) held academic degrees, 2.2% (16/731) held a doctoral degree, and 7.8% (57/731) worked in life sciences-related fields. While our first study was with early adopters who were potentially experts in the field of biology or genetics, this study targeted a much more diverse distribution of people. Users spent just over 12 minutes on average (SD 8) exploring the reports.

#### Comprehension Scores

Comprehension questions assessed users’ abilities to identify variants that indicated increased risk for a particular condition and to understand the certainty of the scientific evidence behind the interpretation. A comprehension score between 0 and 10 was assigned based on users’ responses to nine questions in the form of multiple choice (Table 2; Q3, Q4, Q5, Q7, and Q8), fill in the blank (Table 2; Q1, Q2, and Q6), and select all that apply (Table 2, Q9). There were multiple answers for the “select all that apply” question, which were given separate points. Participants’ abilities to find clinically important variants, both pathogenic and protective, were also assessed. A comparison of the participants’ responses to the comprehension questions across the seven experimental conditions was made using analysis of variance (ANOVA) followed by a post hoc Tukey’s test. Table 6 provides a summary of these findings.

**Table 6:** Summary of comprehension scores across interventions.

Intervention	Average score (SD)
Table (control)	5.65 (1.83)
Bar chart	6.21 (1.56)
Bubble chart	6.30 (1.44)
Treemap	5.74 (1.72)
Heat map	6.25 (1.40)
Zoomable treemap	4.63 (2.16)
Zoomable partitions	5.08 (1.90)

**Figure 12:**
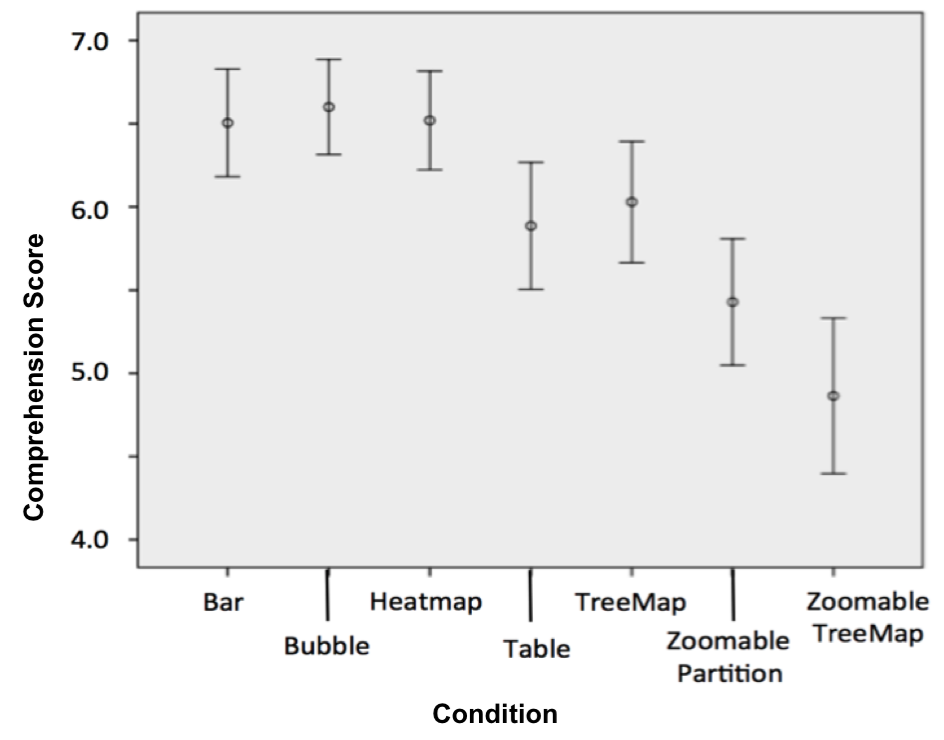
Users' comprehension of the reports across the report types. Error bars represent 95% CI.

The analyses revealed significant differences between the report types (see Figure 12). The zoomable treemap and zoomable partition reports were found to be less effective in communicating genomic data than the visualizations in the other conditions—5.08 and 4.63 out of 10, respectively. Indeed, scores obtained using the zoomable treemap report resulted in significantly lower comprehension scores compared with all nonzoomable reports (*P*<.001), and the zoomable partition report led to significantly lower understanding compared with the bar chart (6.21/10), bubble graph (6.30/10), and heat map reports (6.25/10) (*P*<.001). In addition, the table report (5.65/10) was also found to be significantly less effective than the bubble graph report (*P*=.04).

**Figure 13:**
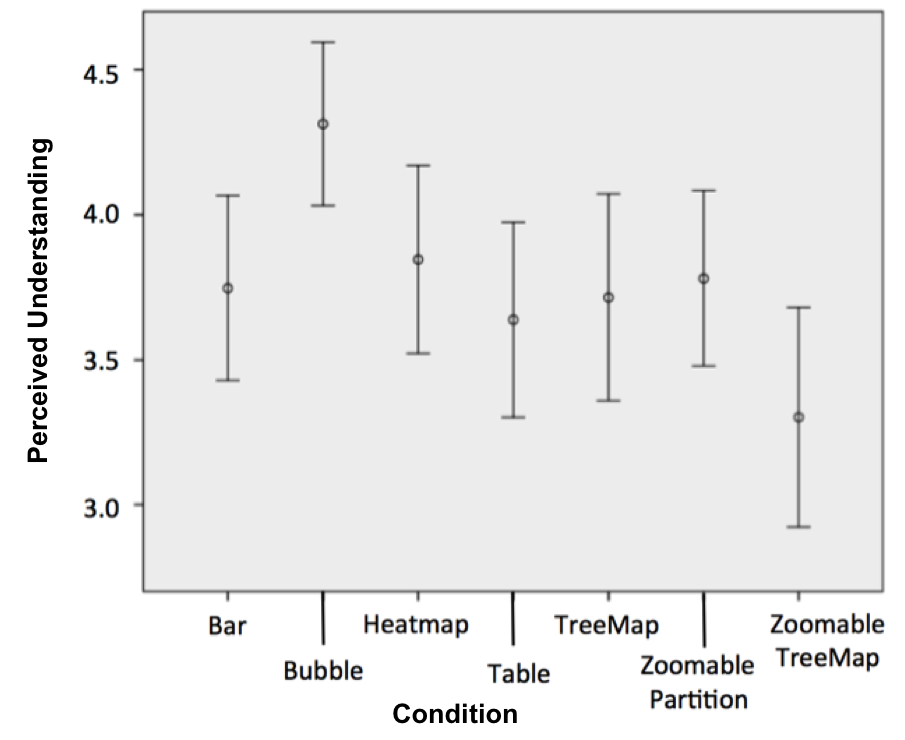
Users' subjective scores of perceived understanding. Error bars represent 95% CI.

Participants were asked to rate their perceived ease of understanding on a 5-point Likert scale (Table 2, Q11). A comparison of responses between the seven report types was made using ANOVA followed by a post hoc Tukey’s test. Analysis demonstrated that perceived understanding was highest in the bubble graph (4.31 out of 5, SD 1.52) and lowest in the zoomable treemap (3.29, SD 1.87) (see Figure 13). The bubble graph was perceived as significantly easier to understand than both the zoomable treemap (3.29, SD 1.87, *P*=.01) and the tabular control condition (3.62, SD 1.74, *P*=.05).

We found significant differences in the results between participants who worked in life science-related fields and others who did not, in terms of both comprehension—where those in the life sciences scored higher—and perceived ease of understanding—where those in the life sciences found it easier to understand. However, when running the statistical analyses comparing the visualization types among people in the life sciences population, the differences between the visualization types were found to be similar to the differences between the visualization types among the entire population.

#### Qualitative Results

Approximately 39.2% (240/612) of participants who responded to the open-ended questions and who were not assigned to the control condition, which did not use color coding, reflected positively on the color-coding scheme. For example, one participant noted that “The color-coded chart made it a lot easier to see the harmful and beneficial genes and how severe they potentially were.” Another participant mentioned, “The use of different colors made some information very easy to see right away.” Only 3.1% (19/612) of participants gave negative feedback on the color coding. About 33.9% (243/716) of all users still found the gene variant summaries too technical and difficult to understand, though 5.7% (41/716) noted that they found the glossary helpful. Approximately 9.8% (60/612) of participants, not including those in the control condition, indicated that presenting information in tooltips when hovering was not effective. Users pointed to trouble navigating and closing the tooltips, as well as maintaining context as the presented tooltip occluded part of the visualization.

Approximately 25.0% (48/192) of the participants in the zoomable visualization intervention (see Figures 8 and 9) commented that they found zooming confusing. For example, one participant described “...too many in and outs...once information is collapsed it's difficult to navigate." Another mentioned, “The information would be easier to understand in some sort of chart or perhaps more than one rather than the interactive diagram. One chart might have a brief overview and the next perhaps more details. I find the diagram frustrating and time-consuming because the information cannot be accessed all at once.” A total of 12.5% (13/104) of participants in the control condition described the ability to sort columns in the table as the most useful feature of the visualization. One user noted that “Being able to change how the information is sorted made it easier to determine what information was important.” The qualitative data did not, however, provide an explanation for why the bubble graph performed so well in the comprehension test.

## Discussion

### Study 1

Findings from Study 1 shed light on information practices and needs of early adopters of personal genomics. We identified preliminary functional requirements for new direct-to-consumer interactive tools for personal genomics including the following: (1) R1: reviewing an annotated report, (2) R2: integrating resources, (3) R3: curating information, (4) R4: comparing genomes, (5) R5: facilitating sharing information, and (6) R6: making content accessible. However, findings from this study also highlighted a need to further investigate detailed elements of interactive genomic reports that were not probed in this study. In particular, expanding our understanding of Task R1, which arguably represents the most basic functionality of existing direct-to-consumer personal genomic tools, in order to understand how users engage with interactive genetic reports to learn about their genomic data. Open-ended questions, which informed Study 2, included the following: How do users approach their personal genomic reports? What makes genomic reports difficult to understand? What features could make genomic information more accessible?

### Study 2

To address these questions, we conducted a workshop with Personal Genome Project participants focusing on their interactions with annotated personal genomic reports. Our goals for this workshop were to gain a more nuanced understanding of how users currently engage with genomic data, how they learn from their own personal genomic data, and what factors impact their understanding and preferences.

Findings indicated that early adopters of personal genomics were motivated by various factors and were not necessarily approaching their personal genomic information to find an answer to a concrete question. Rather, they sought information about gene variants with well-established pathogenic impact and that were of high clinical importance. Results also highlighted factors that make direct-to-consumer genomic reports difficult to understand, including a large amount of textual information, scientific and technical jargon, and the complexity and uncertainty of the interpretation. Finally, several features were found to be potentially helpful in making personal genomic information more accessible and understandable, including the following: (1) presenting a visual summary (eg, a treemap) that highlights important variants based on their clinical importance and potential impact, (2) using nontechnical language and providing a glossary, and (3) allowing users to search and sort the report.

The user population that participated in this study was again consistent with early adopters as described by Rogers’ theory of the diffusion of innovations [37]—users that tend to have advanced education, expert knowledge, and a willingness to engage in trials of new technologies. Thus, many open questions remain about how to make personal genomics accessible to a general nonexpert user population.

As direct-to-consumer genetic testing services become increasingly available to the general public it is important to also study nonexperts’ interactions with genomic reports. Informed by the insights gained in this study with early adopters, we developed several new interactive visual personal genomic reports aimed at nonexperts. Study 3 focused on investigating how alternative visual designs for genomic reports impact nonexpert understanding of personal genomic information.

### Study 3

Findings from this experiment indicate that HCI interventions can improve the understandability of interactive personal genomic reports for a diverse population of consumers compared to existing reports. Findings also highlight the following implications for the design of interactive, visual personal genomic reports:

1. Zoomability might compromise understandability. The findings show that while zoomable reports (see Figures 8 and 9) may provide additional layers of information, they seem to be less effective in conveying personal genomics information to nonexpert users. This may be because such interfaces are less familiar to most nonexpert users, or because a visual summary maintains better context. Offering additional explanation and tutoring may help users to benefit more from such interactive tools.

2. Overview and familiarity. The findings also suggest that nonzoomable report types, which offer an overview of the entire report through a visual summary (ie, bar chart, bubble graph, and heat map), may be better than tables at conveying personal genomic information. Comprehension scores obtained using the bubble graph interface were the only ones to reach statistical significance, but the findings call for more research comparing different report types and interactive features.

3. Comprehension and perceived understandability. Among the report types studied, the bubble-based report combined both high scores of objective understanding—using the comprehension test—and high scores of subjective perceived understandability (see Figures 12 and 13). This finding suggests that this report type is more likely than others to be useful for nonexpert users. Future work will explore the factors that make this report type more effective and preferable than others.

4. Communicating impact and certainty using color. Findings from Study 2 indicated that when exploring their report, users prioritized locating variants with well-established pathogenic impact. The use of color coding, which utilizes both hue and saturation, was found to be effective in helping users to identify high-priority gene variants. Based on the feedback received in Study 2, we chose a three-color, rather than two-color, coding scheme for Study 3—red (pathogenic), white (benign), blue (protective). This color scheme is accessible to users with color vision deficiencies and was found to be effective based on the qualitative results.

5. Hovering and tooltips. In order to simplify the text-heavy tabular design, all six interactive visualizations presented a summary of each gene variant in a tooltip when the user hovered over a gene variant. Findings identified several usability considerations and problems with hovering and tooltips, including (1) the action to trigger the presentation of a tooltip—while deliberate selection limits fluid exploration, hovering may trigger the presentation of tooltips without user intention, (2) visual elements should be large enough to allow the user to hover above a particular element, (3) when displayed, tooltips occluded parts of the visualization, hiding information that was important for maintaining context—several design techniques could be applied to resolve this problem, including semitransparent tooltips, expanding the visualization layer, and presenting information in an alternative area rather than in a tooltip, and (4) what interactive features should be supported by tooltips—participants requested the ability to search within a tooltip, and to save and share the content of tooltips.

### Conclusions and Future Work

We presented findings from three complementing studies that combined qualitative and quantitative methods to inform the design of personal genomic reports. Our findings offer useful insights for designers and researchers interested in the role HCI can play in making personal genomics understandable and useful for consumers. Study 1 explored the information practices and needs of early adopters and identified preliminary functional requirements for new direct-to-consumer personal genomics interactive tools. Extending Study 1 using face-to-face interviews and user demonstrations, Study 2 sought to understand why and how users engage with interactive genetic reports to learn about their personal genomic data. Building on the findings of the first two studies, Study 3 focused on designing and testing alternative interactive reports informed by the needs and practices identified earlier. The designs, using different interactive visualizations, were tested using online experiments with Amazon Mechanical Turk users to investigate how variations in interface design and data visualization affect users’ understanding of, as well as preferences and attitudes toward, personal genomic reports.

To our knowledge, this paper presents the first study that focuses on information practices, requirements, and design considerations for nonexpert engagement with personal genomics. Future work may focus on the role demographic and other personal attributes may have on users’ understanding of different report types. For example, emerging work shows that personality traits are important to how users perceive data visualizations [38]. Understanding how users’ backgrounds and personalities affect their understanding of, and likelihood to act upon, personal genomic reports is important. We also plan to apply findings from the qualitative and experimental research to the design and development of new interactive tools that empower consumers to engage with, and make sense of, their personal genomic information.

## Abbreviations

ANOVA: analysis of variance
API: application programming interface
DIYbio: do-it-yourself biology
DTCGT: direct-to-consumer genetic testing
FDA: Food and Drug Administration
GET: Genomes, Environments, and Traits
HCI: human-computer interaction
HGP: Human Genome Project
HIT: Human Intelligence Task
IIS: Information and Intelligent Systems
NSF: National Science Foundation
PGP: Personal Genome Project
SNP: single nucleotide polymorphism
